# Estimation of maximal lactate steady state using the sweat lactate sensor

**DOI:** 10.1038/s41598-023-36983-8

**Published:** 2023-06-26

**Authors:** Yuki Muramoto, Daisuke Nakashima, Tsubasa Amano, Tomota Harita, Kazuhisa Sugai, Kyohei Daigo, Yuji Iwasawa, Genki Ichihara, Hiroki Okawara, Tomonori Sawada, Akira Kinoda, Yuichi Yamada, Takeshi Kimura, Kazuki Sato, Yoshinori Katsumata

**Affiliations:** 1 Institute for Integrated Sports Medicine, Keio University School of Medicine, Tokyo, Japan; 2 Department of Orthopaedic Surgery, Keio University School of Medicine, Tokyo, Japan; 3 Keio University School of Medicine, Tokyo, Japan; 4 School of Veterinary Nursing and Technology, Faculty of Veterinary Science, Nippon Veterinary and Life Science University, Tokyo, Japan; 5 Department of Cardiology, Keio University School of Medicine, Tokyo, Japan

**Keywords:** Physiology, Medical research

## Abstract

A simple, non-invasive algorithm for maximal lactate steady state (MLSS) assessment has not been developed. We examined whether MLSS can be estimated from the sweat lactate threshold (sLT) using a novel sweat lactate sensor for healthy adults, with consideration of their exercise habits. Fifteen adults representing diverse fitness levels were recruited. Participants with/without exercise habits were defined as trained/untrained, respectively. Constant-load testing for 30 min at 110%, 115%, 120%, and 125% of sLT intensity was performed to determine MLSS. The tissue oxygenation index (TOI) of the thigh was also monitored. MLSS was not fully estimated from sLT, with 110%, 115%, 120%, and 125% of sLT in one, four, three, and seven participants, respectively. The MLSS based on sLT was higher in the trained group as compared to the untrained group. A total of 80% of trained participants had an MLSS of 120% or higher, while 75% of untrained participants had an MLSS of 115% or lower based on sLT. Furthermore, compared to untrained participants, trained participants continued constant-load exercise even if their TOI decreased below the resting baseline (P < 0.01). MLSS was successfully estimated using sLT, with 120% or more in trained participants and 115% or less in untrained participants. This suggests that trained individuals can continue exercising despite decreases in oxygen saturation in lower extremity skeletal muscles.

## Introduction

Exercise with appropriate frequency and intensity is paramount to maintaining good health in all generations and improving exercise performance in athletes. Maximal lactate steady state (MLSS) is the intensity at which constant-workload exercise can be performed for 40–60 min without lactate accumulation^1,2^. Above the MLSS intensity, blood lactate shows an identifiable increase during constant-workload exercise, with a concomitant decrease in oxygen saturation in the vastus lateralis in the thigh^3^. MLSS has been utilized as a measure of training intensity in endurance sports, such as track and field^4^, cycling^5^, and swimming^6,7^. MLSS assessment requires several constant submaximal load tests performed on separate days and frequent blood lactate measurements during exercise, which are complicated and physically strenuous for the participants^8^. Therefore, the lactate threshold (LT) and blood lactate accumulation onset time (OBLA) are frequently used instead of MLSS as measures of training intensity^8^. More specifically, LT and OBLA are thought to reflect low^9^ and high^10^ intensity, respectively, relative to MLSS. However, there are limitations to the use of LT and OBLA for MLSS. Recently, several algorithms for MLSS estimation from LT or OBLA have been developed, mainly for use with athletes^4,11,12^.

Despite being simple indices for determining training intensity, LT and OBLA require frequent blood lactate measurements and exercise cessation to collect blood samples. Since these methods are somewhat invasive, they are impractical, particularly for non-athletes or those with no exercise habits. To overcome this limitation, we developed a sweat lactate sensor for real-time, non-invasive measurement of sweat lactate. The sweat lactate threshold (sLT) is reportedly consistent with the anaerobic metabolic threshold^13^. Therefore, we expected that sLT could be utilized to estimate MLSS with minimal stress on participants. Additionally, this simple and non-invasive algorithm may be applicable to non-athletes as well as athletes.

This study aimed to verify whether MLSS could be estimated from sLT using a sweat lactate sensor for healthy adults, with consideration of their exercise habits, and to investigate whether a decrease in oxygen saturation in the vastus lateralis is a factor contributing to the difference in MLSS based on sLT.

## Results

### In-vitro characterization of the lactate sensor under simulated sweat environments

**Figure 1:**
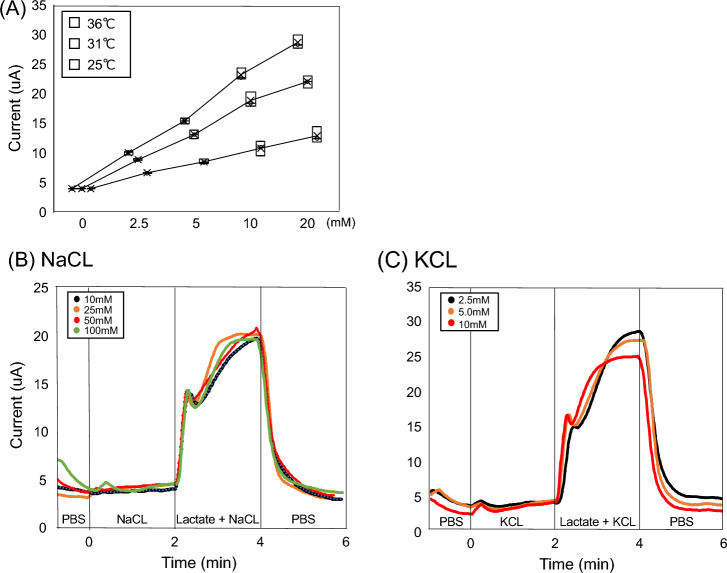
In-vitro characterization of the lactate sensor under imitated sweat environments. (**A**) The graph shows the corresponding calibration plots of the sensor with pH 7 under different temperature (25, 31, and 36 °C) conditions. The interference study for individual lactate (**B,C**). The presence of non-target electrolytes; Na, K, and Cl cause negligible interference to the response of our lactate sensors. Applied voltage = 0.16 V versus Ag/AgCl. The data were obtained from three samples.

Figure 1 showed the amperometric response of the lactate sensor to increasing lactate concentrations in 0, 2.5, 5, 10, and 20 mM. In our sensors, a significant difference was observed in sensor responses to several lactate solutions (2.5, 5, 10, and 20 mM) with different pH (5, 6, 7, and 8) and different temperatures (25, 31, and 36 °C) (Fig. 1A and Online Figs. 1–4). Secondly, a significant response in the sensor to 10 mM lactate solution was observed even in the presence of NaCl (10, 25, 50, 100 mM) and KCl (2.5, 5, 10 mM) (Fig. 1B, C). These findings indicated that the lactate values (current values) of sweat obtained from this sensor could show a significant enough difference to determine the inflection point under various sweat environments.

### Characteristics of participants

The fifteen participants (14 male, 1 female) had a mean age of 26 ± 6 years, mean height of 173 ± 9 cm, mean weight of 67 ± 10 kg, mean skeletal muscle mass of 52 ± 18 kg, mean body fat percentage of 18 ± 7%, mean peak VO_2_ of 41 ± 5 mL/min/kg, and mean HRmax of 175 ± 9 beats per min (bpm). Eleven participants engaged in regular exercise (Table 1).

**Table 1:** Characteristics of participants (mean ± standard deviation).

Measures	All (n = 15)	Trained (n = 11)	Untrained (n = 4)	Mean difference	p-value	95% CI	Cohen’d
Age (year)	26 ± 6	22 ± 4	33 ± 4	−11.3	0.01*	−18.6 to −3.9	2.5
Height (cm)	173 ± 9	173 ± 10	172 ± 7	1.4	0.79	−10.0 to 12.7	0.14
Weight (kg)	67 ± 10	65 ± 8	72 ± 16	−7.6	0.43	−32.1 to 16.9	0.70
Skeletal muscle mass (kg)	52 ± 18	52 ± 8	50 ± 10	1.8	0.76	−12.9 to 16.6	0.21
Body fat percentage (%)	18 ± 7	15 ± 6	25 ± 4	−10.0	0.01*	−16.6 to −3.2	1.59
PeakVO_2_/kg (mL/min/kg)	41 ± 5	43 ± 3	36 ± 6	6.9	0.13	−3.5 to 17.2	1.72
HRmax (bmp)	175 ± 9	174 ± 8	177 ± 7	−2.3	0.61	−12.3 to 7.7	0.27
MLSS (% of sLT)	120 ± 5	122 ± 4	115 ± 4	7.3	0.03*	1.3 to 13.3	1.78
MLSS > 120% (n,%)	10.67%	9.82%	1.25%		0.03*		

P-values refer to the significance of differences between the trained and untrained groups.

MLSS > 120%: number and percentage of participants whose MLSS was 120% or higher.

*bpm* beats per min, *HRmax* maximal heart rate, *peak VO*_*2*_*/kg* peak oxygen uptake/weight, *MLSS* maximal lactate steady state, *sLT* sweat lactate threshold.

*p < 0.05.

### MLSS based on sLT

**Figure 2:**
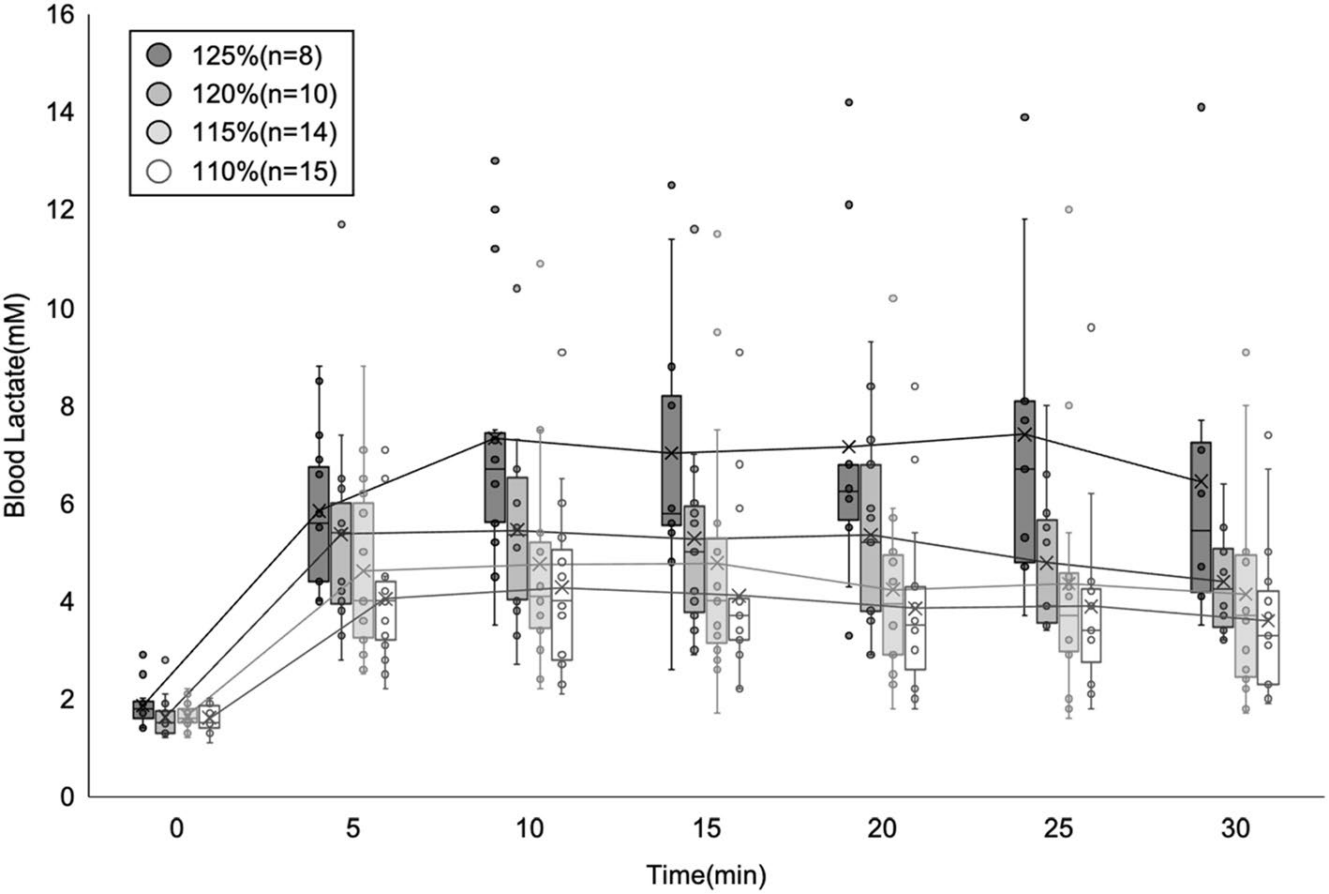
Blood lactate for participants who were able to exercise at each load.

**Figure 3:**
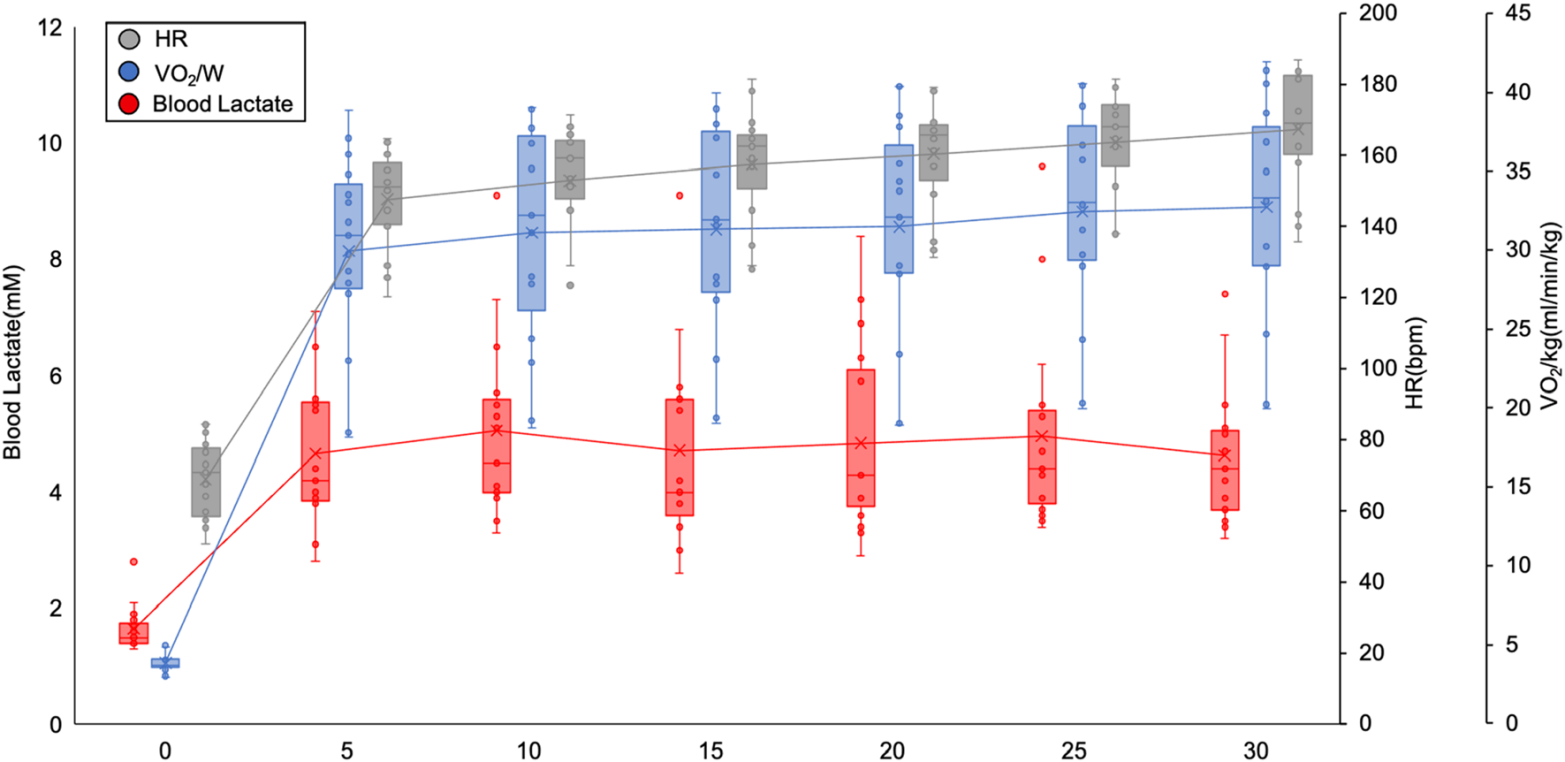
Blood lactate, heart rate, VO_2_/kg at the MLSS in all participants (n = 15). Blood lactate (red), heart rate (gray), and oxygen uptake-adjusted weight (blue) at the MLSS in each participant. *MLSS* maximal lactate steady state, *VO*_*2*_*/kg* oxygen uptake/weight.

The sLT was correlated with the VT (*r* = 0.70) (Online Fig. 5). The Bland–Altman plot described no bias between the mean values (mean differences: −3.0 W, respectively) (Online Fig. 6). Constant-load exercises at 125%, 120%, 115%, and 110% of sLT load were performed in that order, and completed by 8, 10, 14, and 15 participants, respectively (Table 2, Online Fig. 7). Each result shows the values of participants who were able to perform 30 min of exercise. Blood lactate levels at the end of exercise at each load in the aforementioned order were 6.5 ± 3.7 mM, 4.4 ± 1.1 mM, 4.1 ± 1.2 mM, and 3.7 ± 1.7 mM (Table 2, Fig. 2). VO_2_ values (% peak VO_2_) at the end of exercise were 36.3 ± 4.0 mL/min/kg (84.5 ± 7.2%), 32.8 ± 5.5 mL/min/kg (76.9 ± 8.0%), 30.7 ± 6.7 mL/min/kg (71.1 ± 11.1%), and 29.0 ± 6.6 mL/min/kg (68.8 ± 13.1%). HR values (% HRmax) at the end of exercise were 168.6 ± 14.1 bpm (84.8 ± 6.8%), 158.1 ± 14.8 bpm (79.9 ± 7.4%), 152.6 ± 16.2 bpm (77.5 ± 7.8%), and 144.4 ± 15.2 bpm (73.2% ± 7.3%). Among the participants who completed the constant-load exercise at 125% of sLT load, one had an increase in blood lactate > 1 mM at the end of the exercise (30 min). Therefore, MLSS was 125% of sLT in seven participants, 120% in three, 115% in four, and 110% in one, suggesting that estimating MLSS based on sLT was difficult (Table 2). MLSS data obtained from 15 participants were calculated (Table 3, Fig. 3). Blood lactate, % peak VO_2_, and % HRmax at the end of exercise at the MLSS load in the full sample were 4.6 ± 1.3 mM, 77.2 ± 12.7%, and 83.3 ± 6.9%, respectively.

**Table 2:** Exercise data of participants at each load (mean ± standard deviation).

Relative load based on sLT (%)	125%	120%	115%	110%
Exercise completion (n, %)	8 (53%)	10 (66%)	14 (93%)	15 (100%)
MLSS (n, %)	7 (47%)	3 (20%)	4 (27%)	1 (6%)
Measures at the end of the exercise				
Blood lactate (mM)	6.5 ± 4.4	4.4 ± 1.1	4.1 ± 1.2	3.6 ± 1.7
VO_2_/kg (mL/min/kg)	36.3 ± 4.0	32.8 ± 5.5	30.7 ± 6.7	29.0 ± 6.6
% peak VO_2_ (%)	84.5 ± 7.2	76.9 ± 8.0	71.1 ± 11.1	68.8 ± 13.1
HR (bpm)	168.6 ± 14.1	158.1 ± 14.8	152.6 ± 16.2	144.4 ± 15.2
% HRmax (%)	84.8 ± 6.8	79.9 ± 7.4	77.5 ± 7.8	73.2 ± 7.3

Exercise completion: number of participants who were able to achieve 30 min of constant-load exercise.

MLSS: number of participants who were greatest load among the loads in which blood lactate values at the end of exercise (30 min) increased within 1 mM, compared to those at 10 min after exercise initiation.

The value of “measures at the end of the exercise” is exercise completion.

*% HRmax* heart rate/maximal heart rate, *% peak VO*_*2*_ oxygen uptake/peak oxygen uptake, *bpm* beats per min, *HR* heart rate, *MLSS* maximal lactate steady state, *sLT* sweat lactate threshold, *VO*_*2*_*/kg* oxygen uptake/weight.

**Table 3:** Exercise data of participants at MLSS (mean ± standard deviation).

Measures at the end of exercise (n = 15)	
Blood lactate (mM)	4.63 ± 1.21
VO_2_/kg (mL/min/kg)	32.6 ± 7.1
% peak VO_2_ (%)	77.2 ± 12.7
HR (bpm)	164.3 ± 15.4
% HRmax (%)	83.3 ± 6.9
125%:120%:115%:110% (n)	7:3:4:1

*% HRmax* heart rate/maximal heart rate, *% peak VO*_*2*_ oxygen uptake/peak oxygen uptake, *bpm* beats per min, *HR* heart rate, *MLSS* maximal lactate steady state, *VO*_*2*_*/kg* oxygen uptake/weight.

### MLSS based on sLT in participants with daily exercise

Next, the effect of daily exercise on MLSS based on sLT was investigated. The MLSS based on sLT in trained participants was higher than in untrained participants (Table 1). The MLSS for the trained group accounted for more than 120% of the sLT, while the untrained group accounted for less than 115% of the sLT (P = 0.03, φ = 0.55). These findings suggested that MLSS was 120% or more of sLT in regularly trained participants and 115% or less of sLT in untrained participants. To determine a physiological contributor to the difference in MLSS based on sLT between regularly trained and untrained participants, we investigated the relationship between blood lactate accumulation during constant loading tests and a decrease in oxygen saturation in intra-skeletal muscles.

### ΔTOI and blood lactate levels in participants with daily exercise

**Figure 4:**
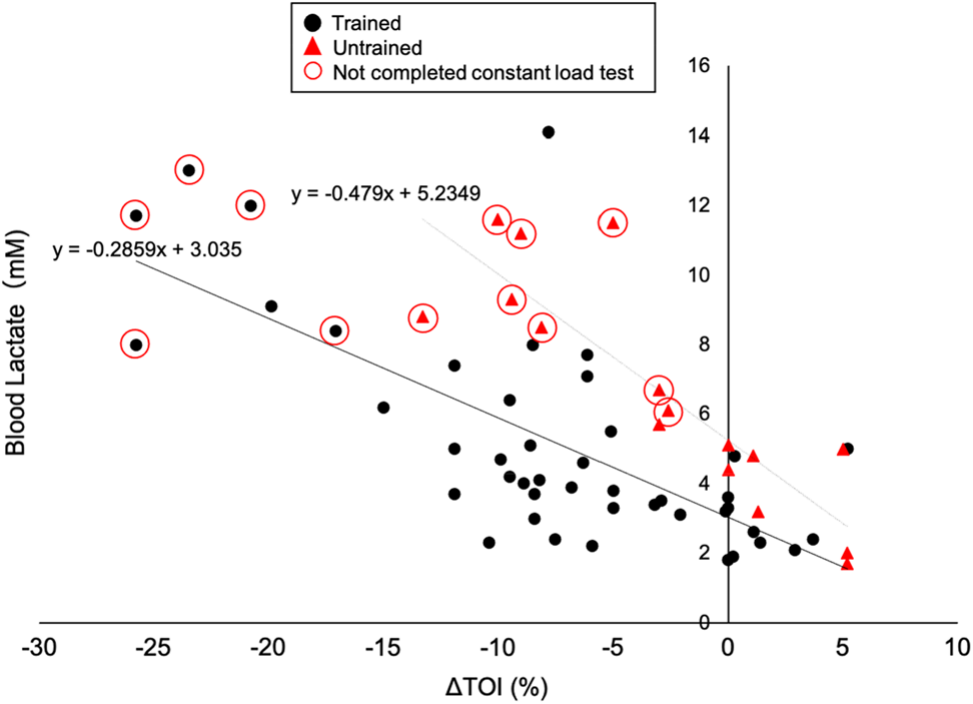
Correlation between change in tissue oxygenation index (TOI) and blood lactate. The black circle represents trained participants, who showed a good correlation between ΔTOI and blood lactate level (y = −0.2859x + 3.035, *r* = −0.7, P < 0.01). The red triangle represents untrained participants, who showed a good correlation between ΔTOI and blood lactate (y = −0.479x + 5.2349, *r* = −0.8, P < 0.01). A steeper increase in blood lactate level was associated with a decrease in ΔTOI in untrained participants as compared to trained participants. *ΔTOI* TOI (pre-post).

**Figure 5:**
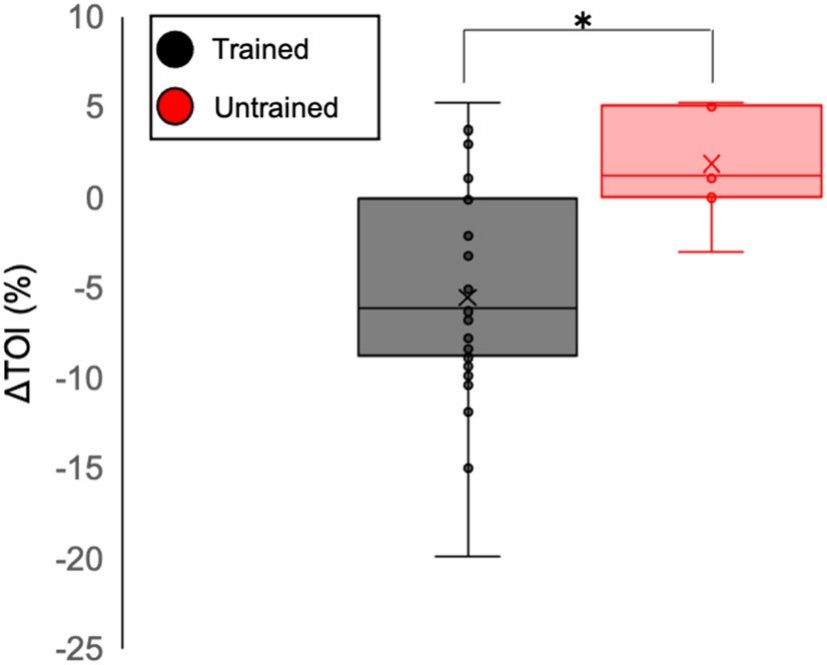
Difference in ΔTOI between trained and untrained participants in the completed exercise. Trained participants continued constant-load exercise even if their ΔTOI decreased (mean difference: −7.4, 95% confidence interval: −11.4 to −3.4, P < 0.01). *ΔTOI* TOI (pre-post); *: P < 0.05.

ΔTOI strongly correlated with blood lactate level at the end of exercise (trained: *r* = −0.7, untrained: *r* = −0.8, Fig. 4 and Online Fig. 8). The plot revealed that the constant-load exercise was discontinued in the untrained group only when ΔTOI was lower than the resting value (Fig. 4, red triangle). In contrast, in regularly trained participants, the exercise continued until up to a 15% decrease in ΔTOI, as compared to the resting value (Fig. 4, black circle). Moreover, a steeper increase in blood lactate was associated with a decrease in ΔTOI in untrained participants as compared to the trained group, suggesting that a slight decrease in ΔTOI immediately contributed to the increase in blood lactate (regression line: trained = −0.286, untrained = −0.479). Further, trained participants continued constant-load exercise even if their ΔTOI decreased (Fig. 5) (mean difference: −7.4, 95% confidence interval [CI]: −11.4 to −3.4, P < 0.01). The optimal cut-off value for completion of the constant-load exercise was estimated to occur at ΔTOI of −17% (sensitivity: 0.97, specificity: 1.00) and −2.6% (sensitivity: 0.88, specificity: 1.00) in trained and untrained participants, respectively, by the ROC curve analysis (Online Fig. 9).

**Figure 6:**
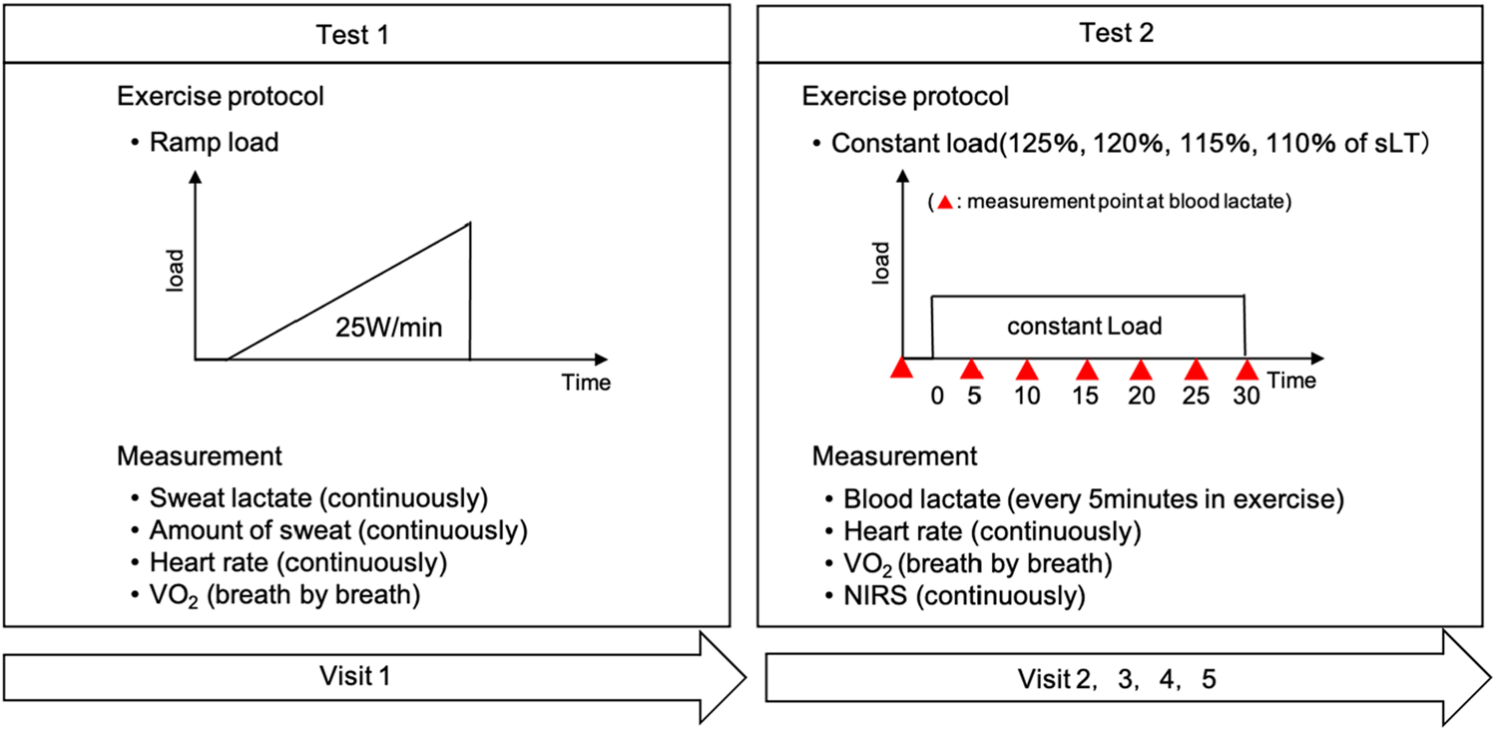
Flowchart of the study protocol. *NIRS* near-infrared spectrometer, *VO*_*2*_ oxygen uptake.

## Discussion

This prospective study provided novel evidence of successful MLSS estimation via sLT calculation by a wearable and non-invasive sweat lactate sensor, with consideration of daily exercise. sLT can be determined independent of the amount of sweating using a sweat lactate sensor on the upper arm^13^. The device also determines the inflection point but not the absolute sweat lactate value^13–15^. The most significant result was that MLSS approximated 120–125% of sLT in regularly trained participants and 115% or less of sLT in untrained participants. The difference in the physiological response to the decrease in oxygen saturation in lower limb skeletal muscle may contribute to this relationship between MLSS and sLT.

Determining MLSS requires multiple constant-load exercise tests. MLSS was defined as the greatest load among the loads in which blood lactate values at the end of exercise (30 min) increased within 1 mM, compared to those at 10 min after exercise initiation^4,11,12^ Therefore, methods have been developed to estimate MLSS using LT and OBLA, with MLSS of 124–127% of LT load^4,11^ and 90% of OBLA load^12^, mainly for athletes^4,11,12^. Additionally, the Functional Threshold Power (FTP) used by cyclists has been determined through several constant submaximal load tests performed on separate days as well as MLSS^16^. FTP is a non-invasive method for measuring training intensity, which correlates well with MLSS^17^. However, LT and OBLA require frequent blood lactate measurements and exercise cessation to collect blood samples. FTP is an index specific to cyclists that requires several constant submaximal load tests performed on separate days and frequent blood lactate measurements during exercise. Therefore, although exercise with appropriate dosage and intensity is essential for maintaining good health in all generations, MLSS measurements are impractical, particularly in non-athletes or those without exercise habits.

A sweat sensor was developed to monitor sweat lactate values in real-time during progressive exercise in a clinical setting and for sports use. Our sensor is highly flexible and can be smoothly adjusted to curved surfaces using PET substrates. The upper arm and forehead are appropriate sites to monitor the lactate levels in sweat due to a high-sweat rate during exercise, smooth skin surfaces for sensor placement, and noninterference during pedaling tasks^13–15^. Especially in healthy subjects, the upper arm has been used because of its simplicity of attachment and minimal interference. sLT defined as the first significant increase in sweat lactate concentration above baseline based on graphical plots, is consistent with LT calculated from blood samples and ventilatory threshold assessed with exhaled gas analysis^13^. In this study, MLSS was successfully estimated via sLT, with 120–125% of sLT in regularly trained participants and 115% or less in untrained participants. Blood lactate, % peak VO_2_, and % HRmax at the end of exercise at MLSS load were consistent with data from previous reports^1,2,8,17,18^. Reportedly, 124–127% of blood LT intensity at the running speed was the MLSS intensity in track and field athletes or cyclists^4,11^. These previous findings were consistent with MLSS load based on sLT in participants who regularly exercised. In untrained participants, MLSS approximated 115% or less of sLT. Assessment of appropriate exercise dosage and intensity should be further targeted for the well-being of non-athletes. MLSS, estimated in a simple and non-invasive manner using a sweat lactate sensor, could be used for health maintenance in non-athletes.

To determine a physiological contributor to the difference in MLSS based on sLT between regularly trained and untrained participants, we investigated the relationship between blood lactate accumulation during constant loading tests and a decrease in oxygen saturation in intra-skeletal muscles. The constant-load exercise was completed for 30 min in trained participants without blood lactate accumulation, even with substantial decreases in oxygen saturation in lower limb skeletal muscles. This finding suggests that training enables constant-load exercise for long periods, even at loads relatively greater than an anaerobic threshold, at which oxygen saturation in intra-skeletal muscles can be preserved. In contrast, a steeper increase in blood lactate was associated with a decrease in ΔTOI in the untrained group as compared to the trained group, suggesting that a slight decrease in ΔTOI immediately contributes to blood lactate accumulation. Exercise tolerance improves through biological responses, such as increased blood flow in skeletal muscles^19^, improved mitochondrial function^20^, and a shift from IIb to IIa in skeletal muscle subsets^21^. These biological responses are induced by the activation of hypoxic response signals following oxygen saturation reduction in skeletal muscles during exercise^22–27^. Therefore, the extent and variability of oxygen saturation reduction during exercise may be related to training effectiveness. Training results in the acquisition of hypoxic tolerance in skeletal muscles, causing increases in exercise endurance and enabling exercise with stronger intensity. Positive feedback between the decrease in oxygen saturation in skeletal muscles and improvement in exercise tolerance could maximize training benefits.

### Limitations

Our findings should be interpreted with consideration of the following limitations. First, because of the observational study design, we cannot exclude the influence of selection bias. Second, our study included a relatively small number of cases, particularly for the untrained group, and primarily healthy college-age male individuals. Further research should include untrained participants and women. Third, constant-load exercises at 130% of sLT load were not performed in this study. Finally, there was a possibility of non-response in the sweat lactate sensor owing to a lack of sweat during exercise. Particularly, older adults and women sweat less^28^. Therefore, in such cases, adjusting exercise parameters to promote sweating is necessary. However, sLT could be clearly determined in all participants in this study.

## Conclusions

By dividing the participants into trained and untrained groups, MLSS was successfully estimated using sLT, with 120% or more of the sLT load in trained participants and 115% or less in untrained participants. This finding may involve the ability of an individual to continue exercising despite a decrease in oxygen saturation in the lower extremity skeletal muscles. This novel actualized measurement of sLT is expected to enable non-invasive MLSS estimation. This simple and non-invasive algorithm can be used as a convenient indicator of good health maintenance for non-athletes and a potential guide for training athletes.

## Methods

### Participants

Fifteen healthy adults representing a broad spectrum of fitness levels, regardless of exercise habits, were recruited between May and September 2022. Participants with/without exercise habits were defined as “trained”, and “untrained,” respectively. Exercise habit was defined as > 75 min per week of exercise at vigorous intensity^29^. The inclusion criteria were as follows: no underlying or pre-existing cardiovascular, respiratory, or metabolic diseases; no athletic injuries; non-smokers; and no dietary supplements or medication habits of any type. The study protocol was approved by the Institutional Review Board of the Keio University School of Medicine (approval number: 20190229) and conducted in accordance with the principles of the Declaration of Helsinki. All participants provided informed consent because the Institutional Review Board approved the use of oral consent, in accordance with the Japanese guidelines for clinical research.

## Experimental procedure

A flowchart of the study protocol is shown in Fig. 6. First, the Ramp stress test was performed using an electromagnetically braked ergometer (StrengthErgo8 V2; Fukuda Denshi Co., Ltd., Tokyo, Japan) with a sweat lactate sensor (Grace Imaging Inc., Tokyo, Japan), an exhaled gas analyzer (Aeromonitor AE-301S; Minato Medical Science Co., Ltd., Osaka, Japan), and a heart rate (HR) monitor (POLAR H10 N; Polar Electro Japan, Tokyo, Japan). Subsequently, constant-load exercise was performed for 30 min at 125%, 120%, 115%, and 110% of sLT intensity in this order. An electromagnetically-braked ergometer was used during the exercise to determine MLSS^12^. At least 24 h were allowed between each test (mean: 7.0 ± 2.9 days)^5^. During constant-load exercise, an exhaled gas analyzer, HR monitor, and near-infrared spectroscopy (NIRS) monitor (NIRO-200NX; Hamamatsu Photonics K.K., Hamamatsu, Japan) were attached. Blood lactate values were obtained via auricular pricking and gentle squeezing of the ear lobe using a blood lactate analyzer (Lactate Pro 2, ARKRAY Inc., Kyoto, Japan). Blood lactate levels were measured before exercise and every 5 min during exercise.

### Exercise test protocol

Participants avoided caffeine and alcohol consumption, which would cause fatigue, the day before testing. After measuring resting data for 2 min, participants performed a warm-up exercise for 2 min at a 50-W load and then exercised at increasing intensities until they could no longer maintain the pedaling rate (volitional exhaustion). The resistance was increased in 25-W increments from 50-W at 1-min intervals. Rotational speed was maintained at 70 rotations per min (rpm).

### sLT determination

A sweat lactate sensor quantifies sweat lactate concentration as a value of current because it reacts with sweat lactate and generates an electric current. The value of current can be obtained as continuous data within 0.1–80 μA in 0.1-μA increments^13^. Further, we investigated whether the lactate values (current values) of sweat obtained from this sensor could show a relative difference significant enough to determine this inflection point under various sweat environments (pH, temperature, and ionic conductivity) with several solutions that were close in composition to actual sweat. Regarding the pH and temperature of human sweat, it has been reported that sweat has a pH of 5–7 and a skin temperature of 25–37 °C^30–32^. Therefore, the electrochemical characterization of the lactate sensor chip was performed using L-lactic acid solutions in 0, 2.5, 5, 10, and 20 mM prepared in 0.1 mol/L phosphate buffer solution (PBS) under different temperatures (25, 31, 36 °C) and pH (5, 6, 7, and 8). Then, the three lactate sensor tips were evaluated in each condition using chronoamperometry at an applied voltage of 0.16 V (versus Ag/AgCl). Next, the major electrolytes in sweat are Na, K, and Cl. Generally, NaCl varies from 10 to 90 mM and KCl from 2 to 8 mM during exercise^30^. Therefore, the sensor evaluated a significant response to l-lactic acid solution in 10 mM even in the presence of NaCl (10, 25, 50, 100 mM) and KCl (2.5, 5, 10 mM).

**Figure 7:**
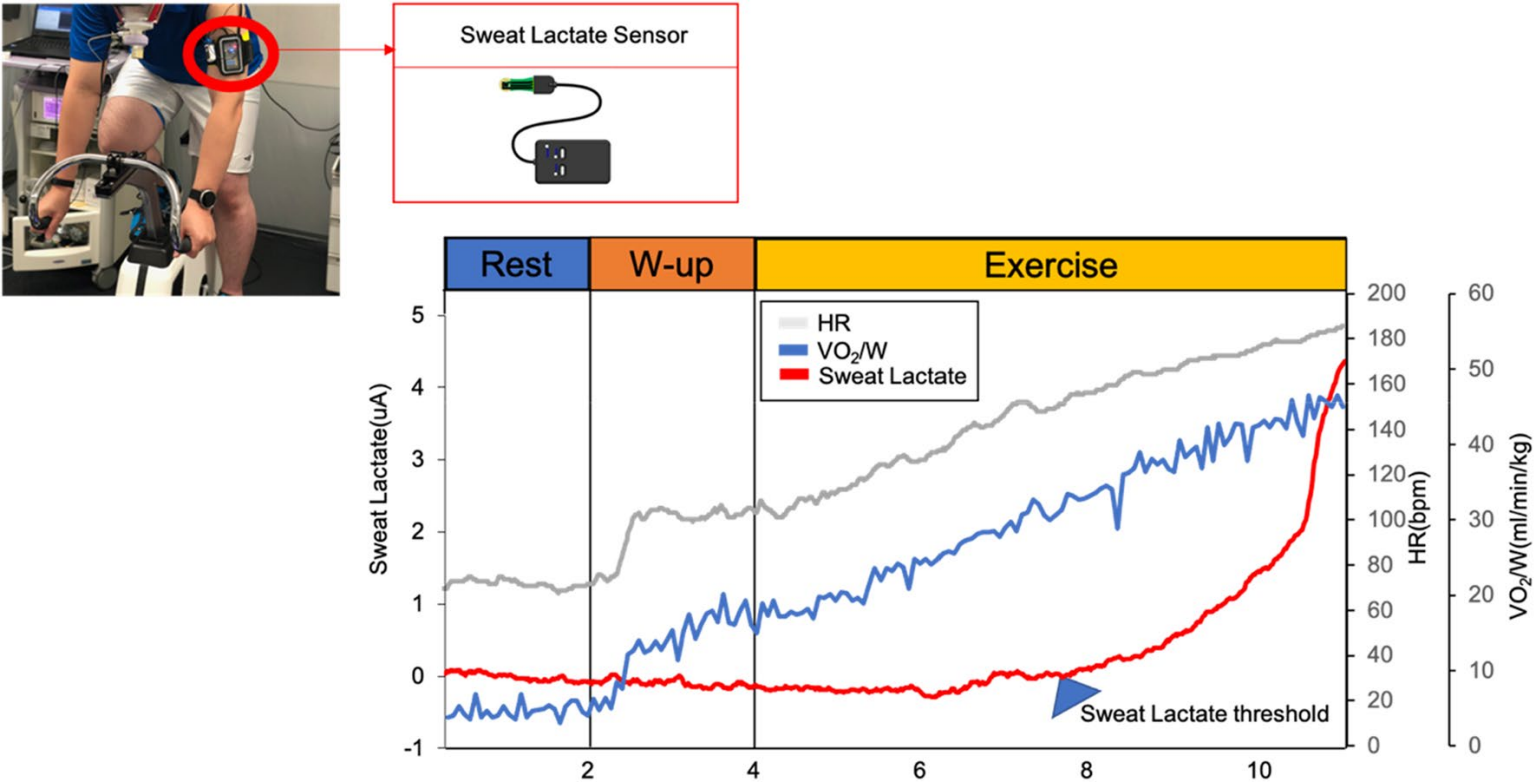
Sweat lactate levels during ramp exercise. *HR* heart rate, *VO*_*2*_*/W* oxygen uptake/weight.

After calibration using saline for 2 or 3 min, the sensor chip connected to the sensor device was attached to the superior right upper limb of the participant^13,14^, which was cleaned with an alcohol-free cloth. The upper arm has a high-sweat rate during physical excursions^33^. In addition, it is a site that does not interfere with exercise during pedaling tasks. Additionally, data were recorded at a 1-Hz sampling frequency for mobile applications with a Bluetooth connection. Recorded data were converted to moving average values over 13-s intervals and individually underwent zero correction using the baseline value. sLT was defined as the first significant increase in sweat lactate concentration above baseline based on a graphical plot (Fig. 7)^13–15,34^.

### MLSS determination

**Figure 8:**
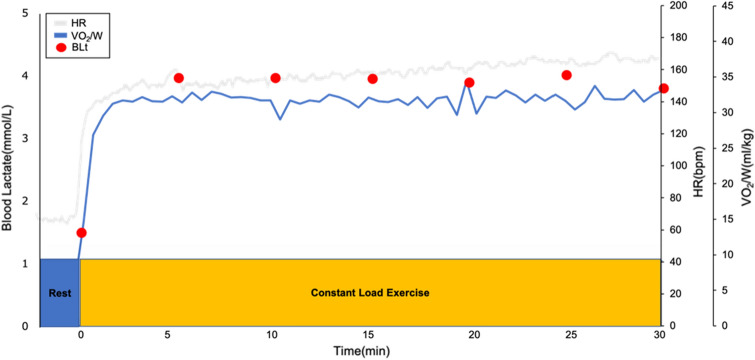
Imaging of the constant-load exercise. *HR* heart rate, *VO*_*2*_*/W* oxygen uptake/weight, *BLt* blood lactate.

Blood lactate was measured before exercise and every 5 min during constant-load exercise for 30 min at 110%, 115%, 120%, and 125% of sLT intensity. The rotational speed was set at 70 rpm. The criteria that did not achieve the exercise and exceeded the MLSS included participants who could not finish the trial due to fatigue, but could not maintain bicycle pedaling at 70 rpm, as well as participants who could finish 30 min of exercise but had an increase in blood lactate of more than 1 mM from 10 min after exercise initiation to the end of the exercise. MLSS was defined as the greatest load among the loads in which blood lactate values at the end of exercise (30 min) increased within 1 mM, compared to those at 10 min after exercise initiation^12^ (Fig. 8).

### Measurement data

On the first day of measurement, body weight, body fat, and skeletal muscle mass were measured using In-Body (InBody470; InBody Japan Inc., Tokyo, Japan). Expired gas flow was measured using a breath-by-breath automated system. Three calibration processes were performed on the system: flow volume sensor, gas analyzer, and delay time calibration. Parameters of respiratory gas exchange, including ventilation (VE), oxygen uptake (VO_2_), and carbon dioxide production (VCO_2_), were continuously monitored and measured using a 10-s average. Skeletal muscle oxygenation in the right thigh was measured using NIRS spectroscopy. The monitor consists of a light-sending probe and a light-receiving probe. Near-infrared light emitted from the light-sending unit is absorbed by skeletal muscle tissue, and changes in the intensity of the light returned to the light-receiving unit enable tissue oxygenation measurement^35^. A pair of probes was attached 4 cm apart on the skin over the vastus lateralis muscle in the distal third of the thigh^36,37^ and then covered and secured with tape^38^. In this study, tissue hemoglobin oxygen saturation (tissue oxygenation index [TOI]), calculated using the spatially resolved spectroscopy method, was assessed^39,40^.

### Statistical analyses

All data are presented as means and standard deviations. The obtained HR and VO_2_ were calculated as a percentage of the maximal HR (% HRmax) and peak VO_2_ (% peak VO_2_). The relationships between the sLT and ventilatory threshold (VT) were investigated using Pearson's correlations. Additionally, the Bland and Altman technique was applied to verify the similarities among the different methods. This comparison is a graphical representation of the difference between the methods and the average of these methods. As previous reports have shown that MLSS is 120% or more of LT intensity, we divided our cohort into two groups using the cut-off of 120% of sLT intensity^4,11,12^. Unpaired *t*-tests and Chi-squared tests were used to compare participant characteristics between the two groups.

The correlation value was used to determine the relationship between the relative change in TOI from baseline (ΔTOI) and blood lactate at the end of the exercise. Unpaired *t*-tests were used to compare ΔTOI across trained and untrained participants.

Receiver operating characteristic (ROC) curve analysis was used to determine the ΔTOI cut-off value for the completed constant exercise test. All analyses were performed using SPSS version 28 software (IBM Japan Ltd., Tokyo, Japan). Statistical significance was set at P < 0.05.

## Supplementary Information

Supplementary Figures.

## Data Availability

All data from these studies are contained within this manuscript or are available from the corresponding author upon reasonable request. Source data are provided in this paper.

## References

[1] Faude O, Kindermann W, Meyer T (2009). Lactate threshold concepts: How valid are they?. Sports Med..

[2] Beneke R (2003). Methodological aspects of maximal lactate steady state-implications for performance testing. Eur. J. Appl. Physiol..

[3] Azevedo RA, Forot J, Millet GY, Murias JM (2022). Comparing of muscle V̇O_2_ from near-infrared spectroscopy desaturation rate to pulmonary V̇O_2_ during cycling below, at, and above the maximal lactate steady state. J. Appl. Physiol..

[4] Garcia-Tabar I, Gorostiaga EM (2018). A “blood relationship” between the overlooked minimum lactate equivalent and maximal lactate steady state in trained runners. Back to the old days?. Front. Physiol..

[5] Greco CC, Barbosa LF, Caritá RA, Denadai BS (2012). Is maximal lactate steady state during intermittent cycling different for active compared with passive recovery?. Appl. Physiol. Nutr. Metab..

[6] Pelarigo JG, Machado L, Fernandes RJ, Greco CC, Vilas-Boas JP (2017). Oxygen uptake kinetics and energy system’s contribution around maximal lactate steady state swimming intensity. PLoS ONE.

[7] Espada MC (2015). Ventilatory and physiological responses in swimmers below and above their maximal lactate steady state. J. Strength. Cond. Res..

[8] Jones AM, Burnley M, Black MI, Poole DC, Vanhatalo A (2019). The maximal metabolic steady state: Redefining the “gold standard”. Physiol. Rep..

[9] Kilding AE, Jones AM (2005). Validity of a single-visit protocol to estimate the maximum lactate steady state. Med. Sci. Sports Exerc..

[10] Baldari C, Guidetti L (2000). A simple method for individual anaerobic threshold as predictor of max lactate steady state. Med. Sci. Sports Exerc..

[11] Garcia-Tabar I, Rampinini E, Gorostiaga EM (2019). Lactate equivalent for maximal lactate steady state determination in soccer. Res. Q. Exerc. Sport.

[12] Urhausen A, Coen B, Weiler B, Kindermann W (1993). Individual anaerobic threshold and maximum lactate steady state. Int. J. Sports Med..

[13] Seki Y (2021). A novel device for detecting anaerobic threshold using sweat lactate during exercise. Sci. Rep..

[14] Maeda Y (2023). Implications of the onset of sweating on the sweat lactate threshold. Sensors.

[15] Katsumata Y (2021). Laminar flow ventilation system to prevent airborne infection during exercise in the COVID-19 crisis: A single-center observational study. PLoS ONE.

[16] Bräuer EK, Smekal G (2020). VO_2_ steady state at and just above the maximum lactate steady state intensity. Int. J. Sports Med..

[17] Iannetta D (2020). A critical evaluation of current methods for exercise prescription in women and men. Med. Sci. Sports Exerc..

[18] Vobejda C, Fromme K, Samson W, Zimmermann E (2006). Maximal constant heart rate—A heart rate based method to estimate maximal lactate steady state in running. Int. J. Sports Med..

[19] Egan B, Zierath JR (2013). Exercise metabolism and the molecular regulation of skeletal muscle adaptation. Cell Metab..

[20] Hood DA, Memme JM, Oliveira AN, Triolo M (2019). Maintenance of skeletal muscle mitochondria in health, exercise, and aging. Annu. Rev. Physiol..

[21] Wilson JM (2012). The effects of endurance, strength, and power training on muscle fiber type shifting. J. Strength Cond. Res..

[22] Gatterer HE (2018). Exercise performance, muscle oxygen extraction and blood cell mitochondrial respiration after repeated-sprint and sprint interval training in hypoxia: A pilot study. J. Sports Sci. Med..

[23] Lindholm ME, Rundqvist H (2016). Skeletal muscle hypoxia-inducible factor-1 and exercise. Exp. Physiol..

[24] Marshall HC (2008). Effects of intermittent hypoxia on SaO_2_, cerebral and muscle oxygenation during maximal exercise in athletes with exercise-induced hypoxemia. Eur. J. Appl. Physiol..

[25] Nagahisa H, Mukai K, Ohmura H, Takahashi T, Miyata H (2016). Effect of high-intensity training in normobaric hypoxia on thoroughbred skeletal muscle. Oxid. Med. Cell Longev..

[26] Pramkratok W, Songsupap T, Yimlamai T (2022). Repeated sprint training under hypoxia improves aerobic performance and repeated sprint ability by enhancing muscle deoxygenation and markers of angiogenesis in rugby sevens. Eur. J. Appl. Physiol..

[27] Suzuki J (2016). Short-duration intermittent hypoxia enhances endurance capacity by improving muscle fatty acid metabolism in mice. Physiol. Rep..

[28] D’Souza AW, Notley SR, Kenny GP (2020). The relation between age and sex on whole-body heat loss during exercise-heat stress. Med. Sci. Sports Exerc..

[29] Bull FC (2020). World Health Organization 2020 guidelines on physical activity and sedentary behaviour. Br. J. Sports Med..

[30] Baker LB, Wolfe AS (2020). Physiological mechanisms determining eccrine sweat composition. Eur. J. Appl. Physiol..

[31] Mehnert P (2000). Prediction of the average skin temperature in warm and hot environments. Eur. J. Appl. Physiol..

[32] Torii M, Yamasaki M, Sasaki T, Nakayama H (1992). Fall in skin temperature of exercising man. Br. J. Sports Med..

[33] Havenith G, Fogarty A, Bartlett R, Smith CJ, Ventenat V (2008). Male and female upper body sweat distribution during running measured with technical absorbents. Eur. J. Appl. Physiol..

[34] Okawara H (2022). Kinetic changes in sweat lactate following fatigue during constant workload exercise. Physiol. Rep..

[35] Grassi B (2003). Muscle oxygenation and pulmonary gas exchange kinetics during cycling exercise on-transitions in humans. J. Appl. Physiol..

[36] Ishii K (2012). Central command contributes to increased blood flow in the noncontracting muscle at the start of one-legged dynamic exercise in humans. J. Appl. Physiol..

[37] Ishii K (2016). Central command generated prior to arbitrary motor execution induces muscle vasodilatation at the beginning of dynamic exercise. J. Appl. Physiol..

[38] Kowalchuk JM, Rossiter HB, Ward SA, Whipp BJ (2002). The effect of resistive breathing on leg muscle oxygenation using near-infrared spectroscopy during exercise in men. Exp. Physiol..

[39] Grassi B, Quaresima V (2016). Near-infrared spectroscopy and skeletal muscle oxidative function in vivo in health and disease: A review from an exercise physiology perspective. J. Biomed. Opt..

[40] Kurihara K, Kikukawa A, Kobayashi A, Nakadate T (2007). Frontal cortical oxygenation changes during gravity-induced loss of consciousness in humans: A near-infrared spatially resolved spectroscopic study. J. Appl. Physiol..

