# Supplementary material for: Estimation of maximal lactate steady state using the sweat lactate sensor
Source: Sci Rep. 2023 Jun 26;13:10366. doi: 10.1038/s41598-023-36983-8 (PMC10293173; doi:10.1038/s41598-023-36983-8)
Supplement: Supplementary file 1 — Supplementary Figures. [file 41598_2023_36983_MOESM1_ESM.docx]

Supplemental Material

**Title: Estimation of Maximal Lactate Steady State using sweat lactate sensor**

**Authors:** Yuki Muramoto, Daisuke Nakashima, Tsubasa Amano, Tomota Harita, Kazuhisa Sugai, Daigo Kyohei, Yuji Iwasawa, Genki Ichihara, Hiroki Okawara, Tomonori Sawada, Akira Kinoda, Yuichi Yamada, Takeshi Kimura, Kazuki Sato, Yoshinori Katsumata

This supplemental material has been provided by the authors to give readers additional information about their work.


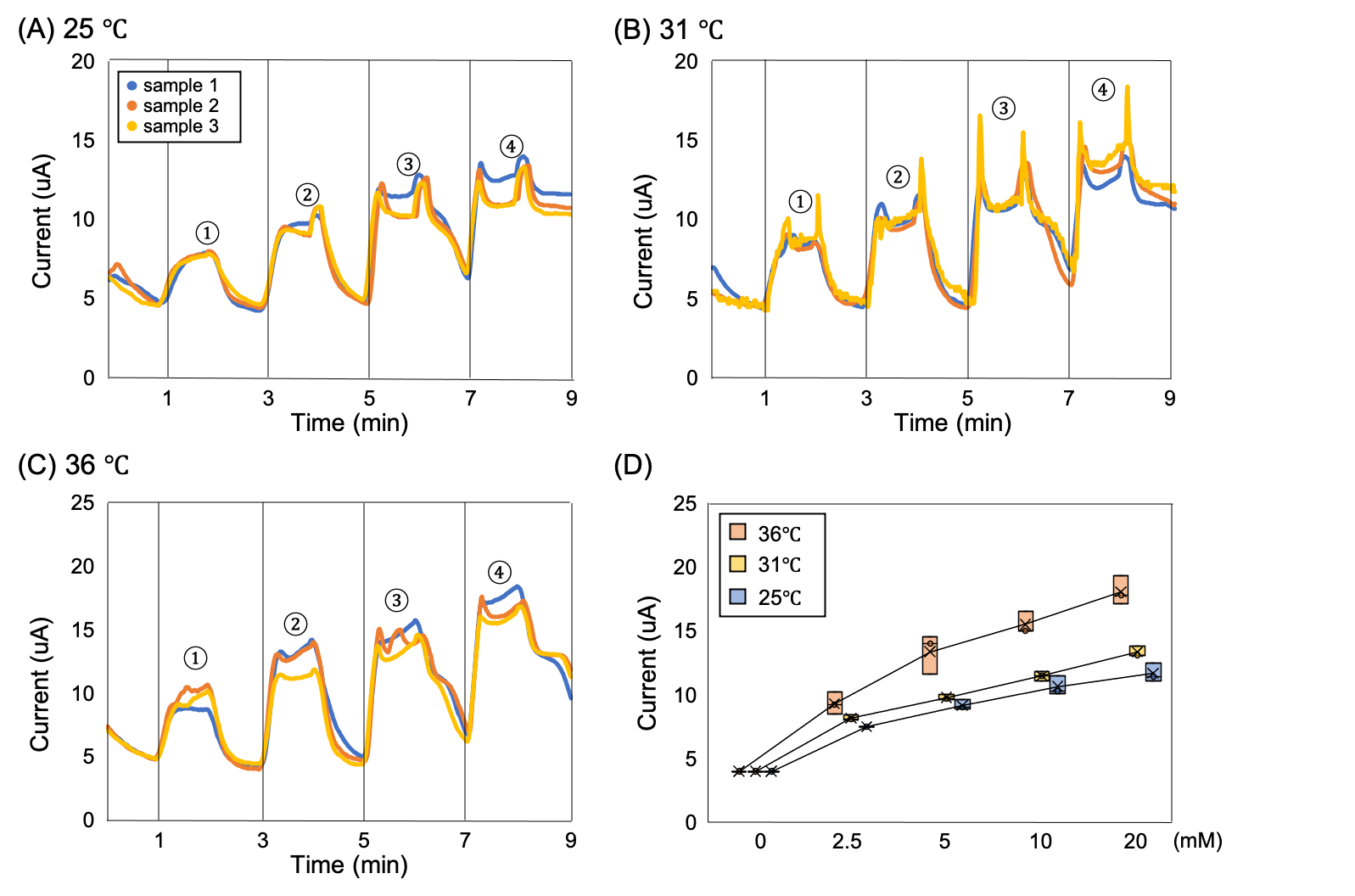


**Online Supplemental Figure 1. Response to L-lactic acid solution adjusted to 0, 2.5, 5, 10, and 20 mM in phosphate buffer (pH 5)**

The graph shows amperometric response to L-lactic acid solution adjusted to 0, 2.5, 5, 10, and 20 mM in phosphate buffer (pH 5) under (A) 25℃, (B) 31℃, (C) 36℃ (①: 2.5, ②: 5, ③: 10, and ④: 20 mM). (D) the graph shows the corresponding calibration plots of the sensor. Applied voltage=0.16 V versus Ag/AgCl. The data were obtained from three samples.


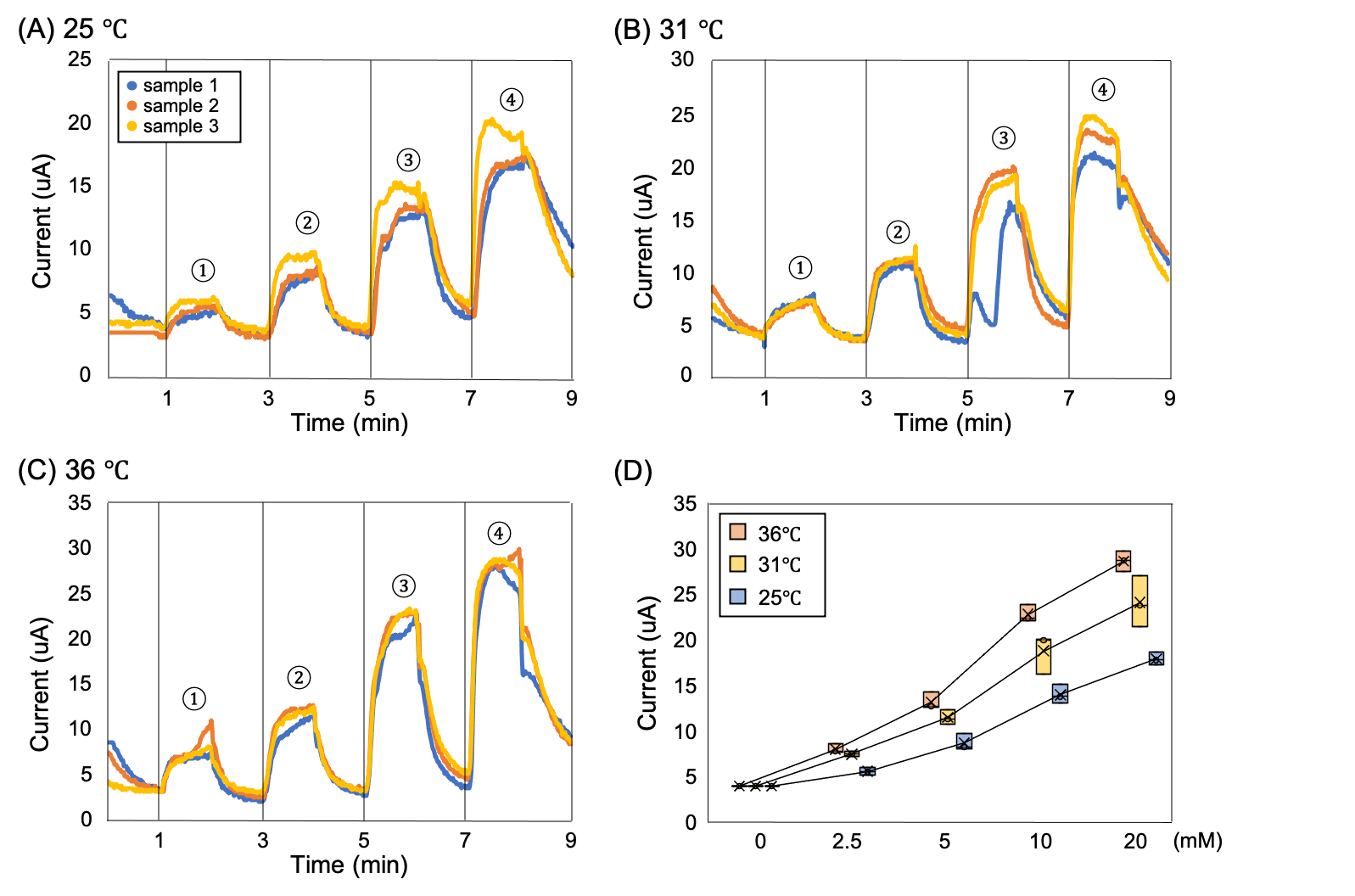


**Online Supplemental Figure 2. Response to L-lactic acid solution adjusted to 0, 2.5, 5, 10, and 20 mM in phosphate buffer (pH 6)**

The graph shows amperometric response to L-lactic acid solution adjusted to 0, 2.5, 5, 10, and 20 mM in phosphate buffer (pH 6) under (A) 25℃, (B) 31℃, (C) 36℃ (①: 2.5, ②: 5, ③: 10, and ④: 20 mM). (D) the graph shows the corresponding calibration plots of the sensor. Applied voltage=0.16 V versus Ag/AgCl. The data were obtained from three samples.


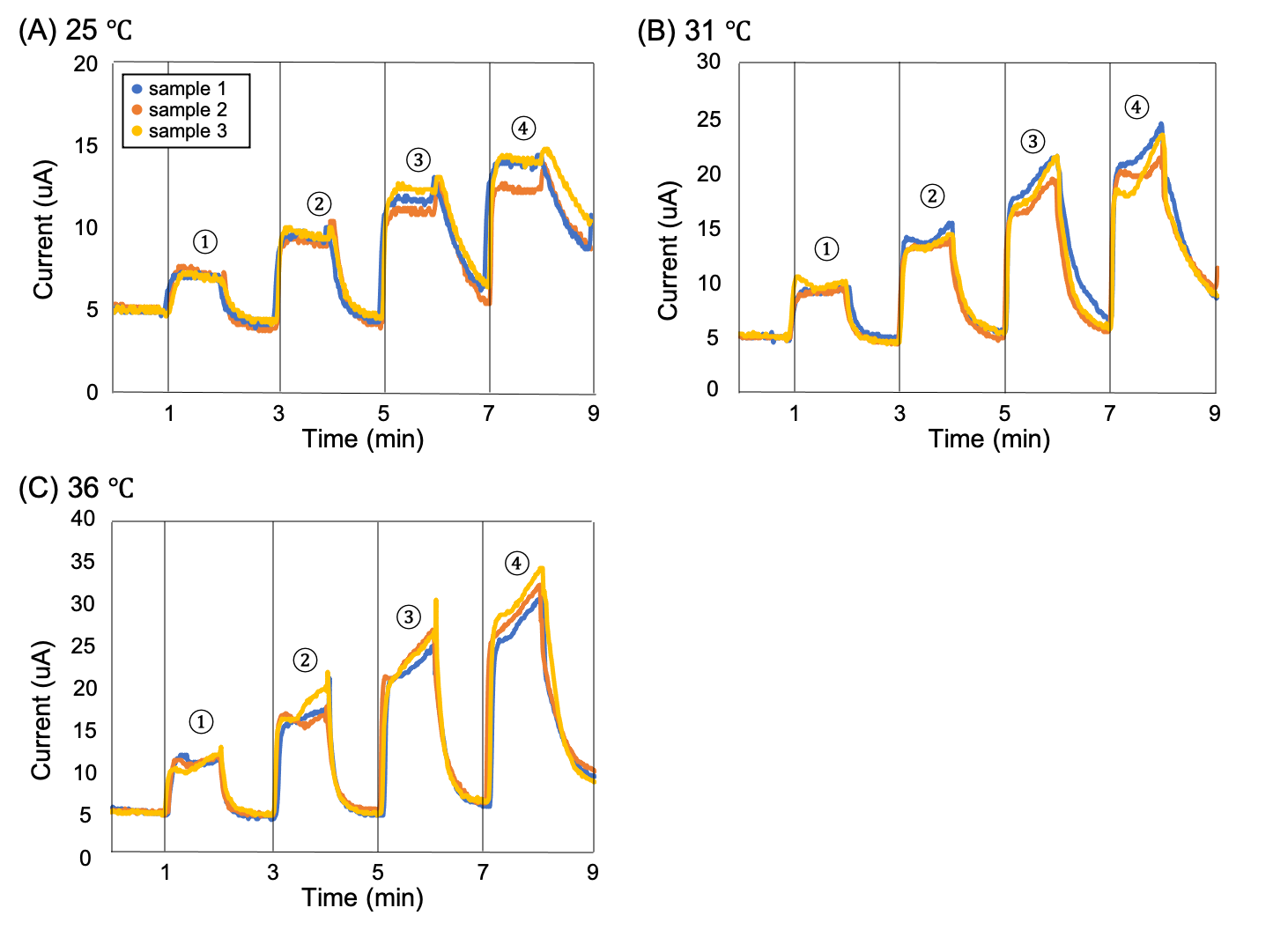


**Online Supplemental Figure 3. Response to L-lactic acid solution adjusted to 0, 2.5, 5, 10, and 20 mM in phosphate buffer (pH 7)**

The graph shows amperometric response to L-lactic acid solution adjusted to 0, 2.5, 5, 10, and 20 mM in phosphate buffer (pH 7) under (A) 25℃, (B) 31℃, (C) 36℃ (①: 2.5, ②: 5, ③: 10, and ④: 20 mM). Applied voltage=0.16 V versus Ag/AgCl. The data were obtained from three samples.


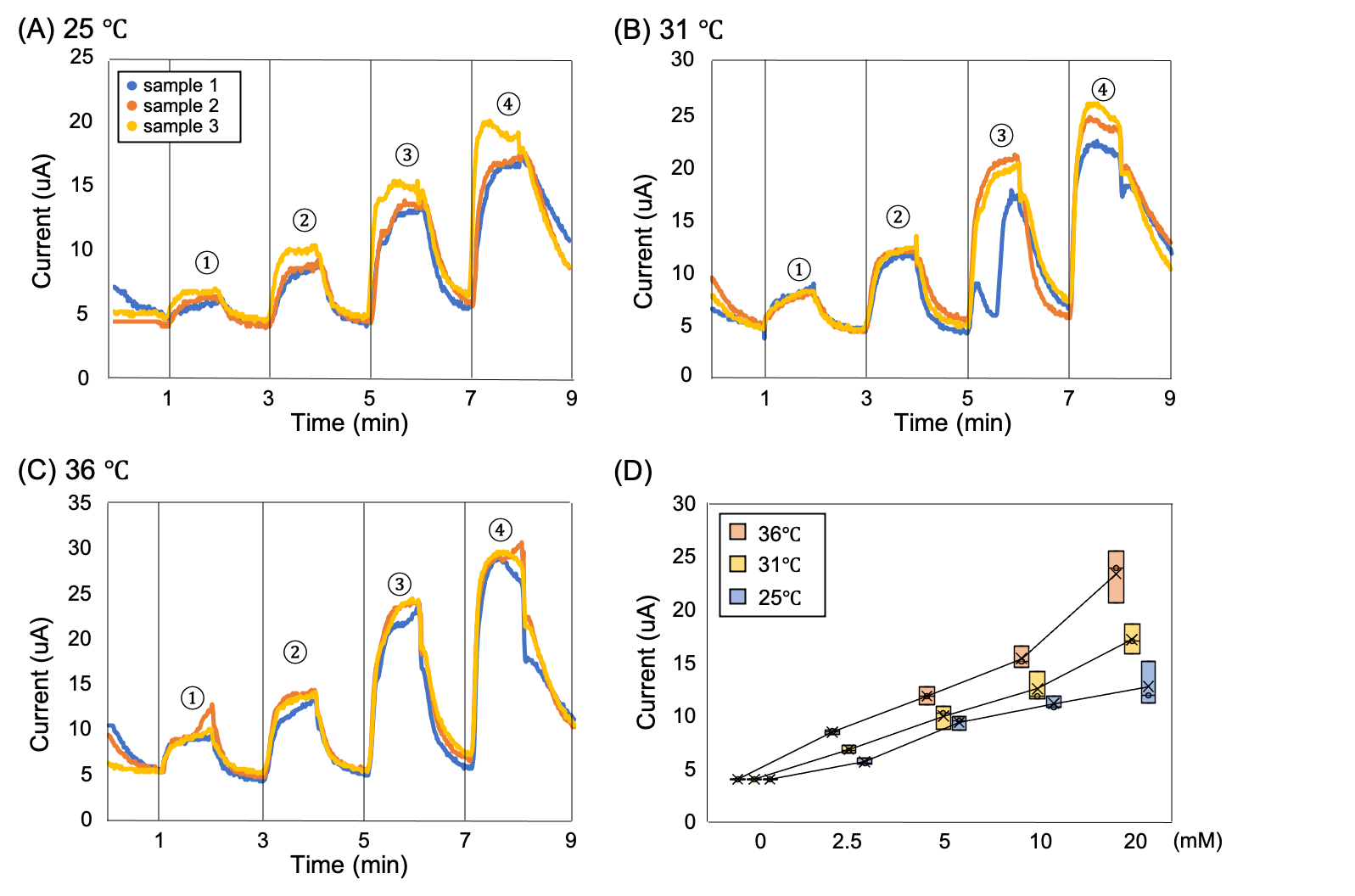


**Online Supplemental Figure 4. Response to L-lactic acid solution adjusted to 0, 2.5, 5, 10, and 20 mM in phosphate buffer (pH 8)**

The graph shows amperometric response to L-lactic acid solution adjusted to 0, 2.5, 5, 10, and 20 mM in phosphate buffer (pH 8) under (A) 25℃, (B) 31℃, (C) 36℃ (①: 2.5, ②: 5, ③: 10, and ④: 20 mM). (D) the graph shows the corresponding calibration plots of the sensor. Applied voltage=0.16 V versus Ag/AgCl. The data were obtained from three samples.


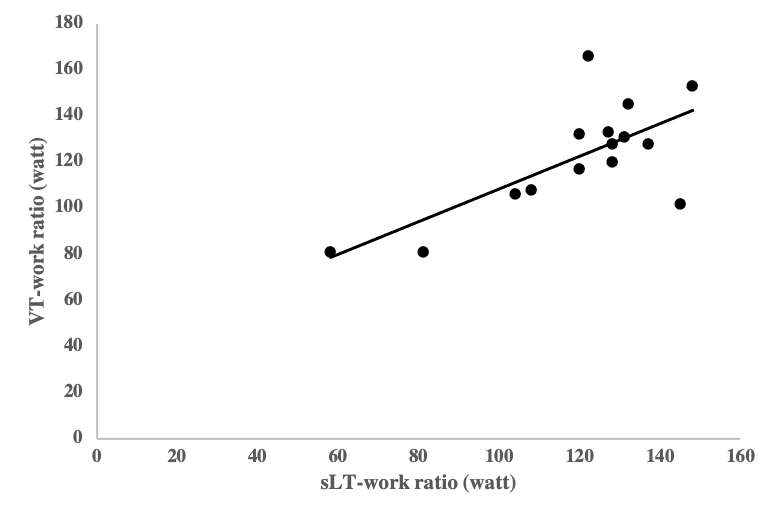


**Online Supplemental Figure 5: Correlation between work ratio at sweat lactate threshold and ventilation threshold.**

The work ratio at sweat lactate threshold was well correlated with work ratio at ventilation threshold(r=0.70, p < 0.01).


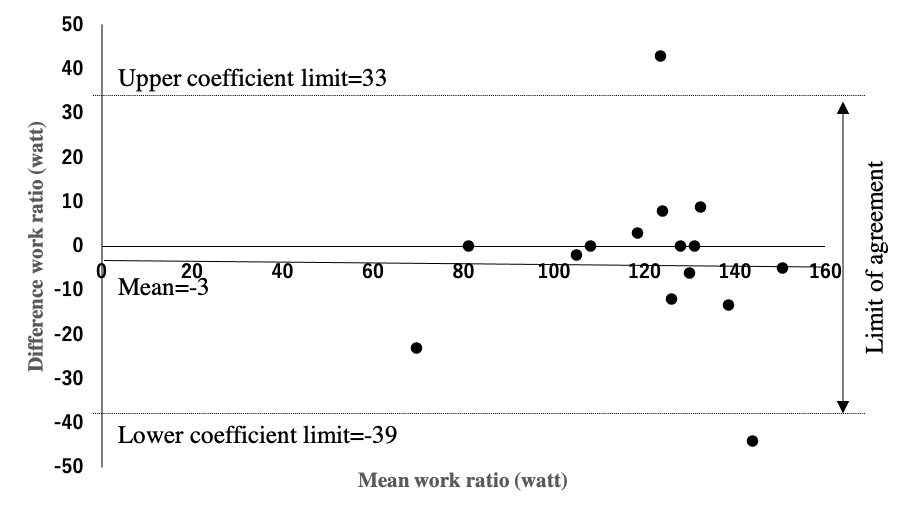


**Online Supplemental Figure 6: Bland-Altman plot load at sweat lactate threshold and load at ventilation threshold.**

This graph shows the scatter plot between the difference and average in the work ratio at ventilation threshold

and sweat lactate threshold. The fixed bias was denied based on 95% coefficient interval of the difference between

the work ratio at ventilation threshold and sweat lactate threshold (Mean difference=-3, 95%CI: -39 ~ 33).

Similarly, as there was no significant correlation bias was not clear.


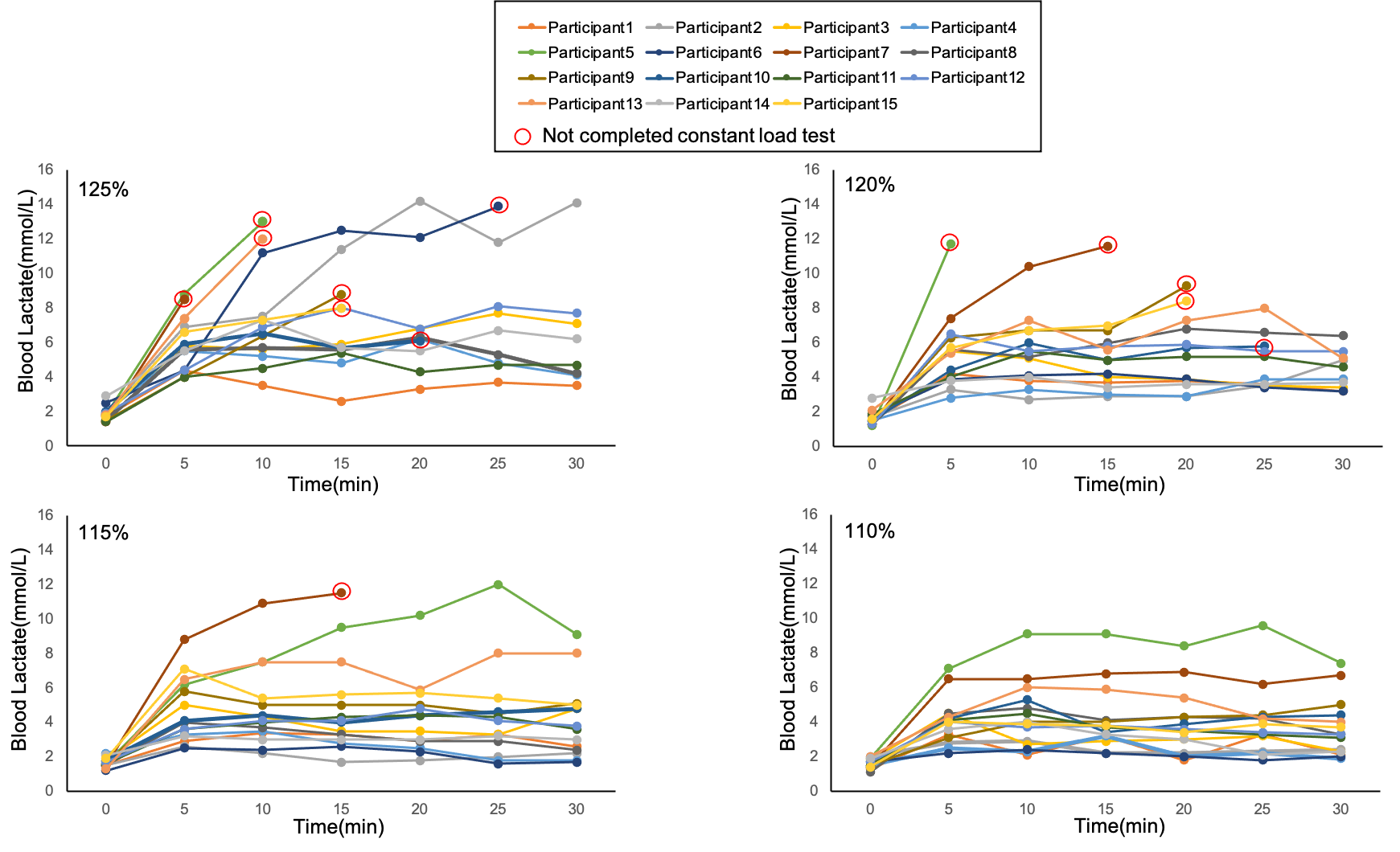


**Online Supplemental Figure 7: Constant load test results at each load.**


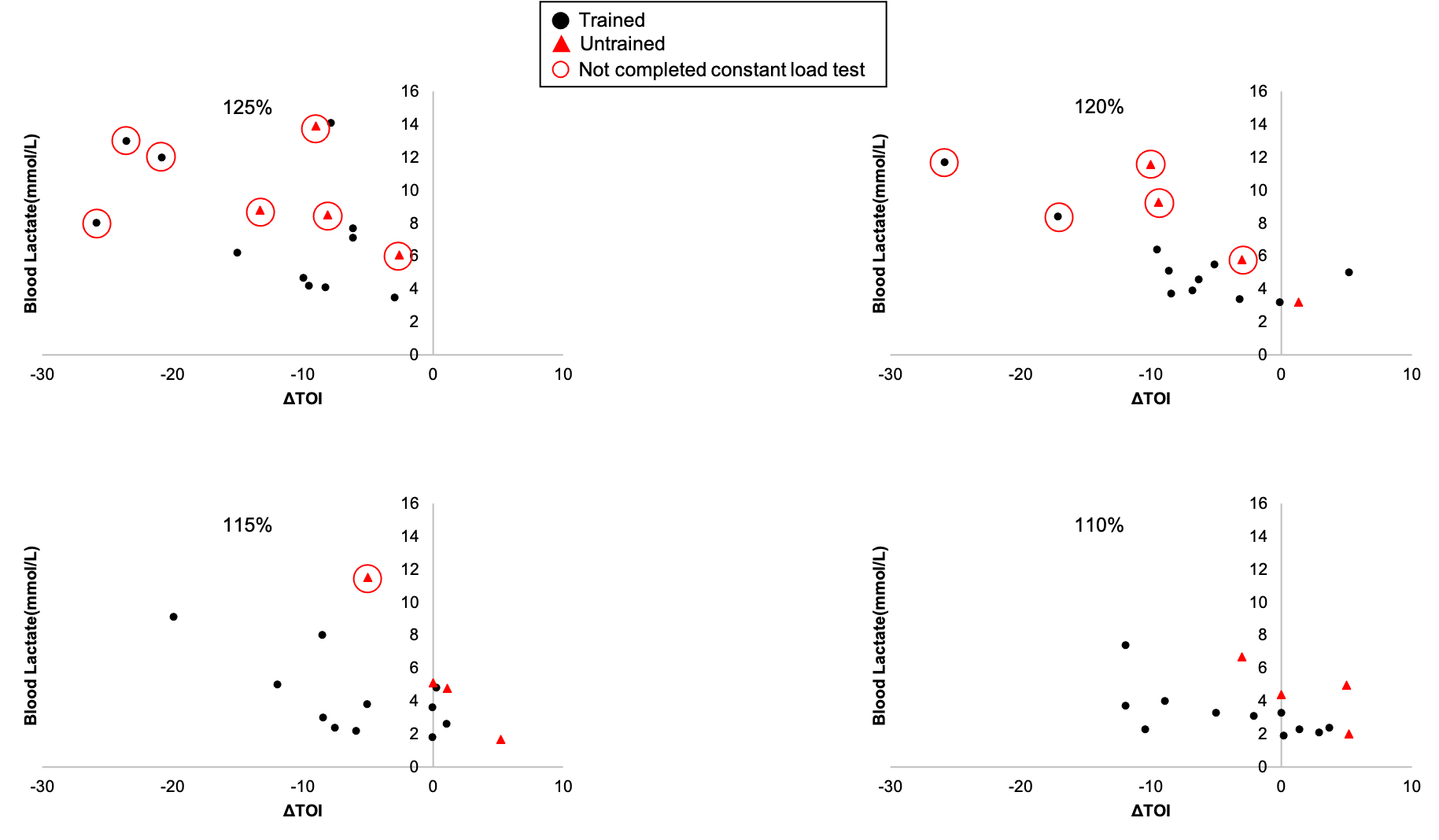


**Online Supplemental Figure 8: Correlation between change in TOI and blood lactate at each load**


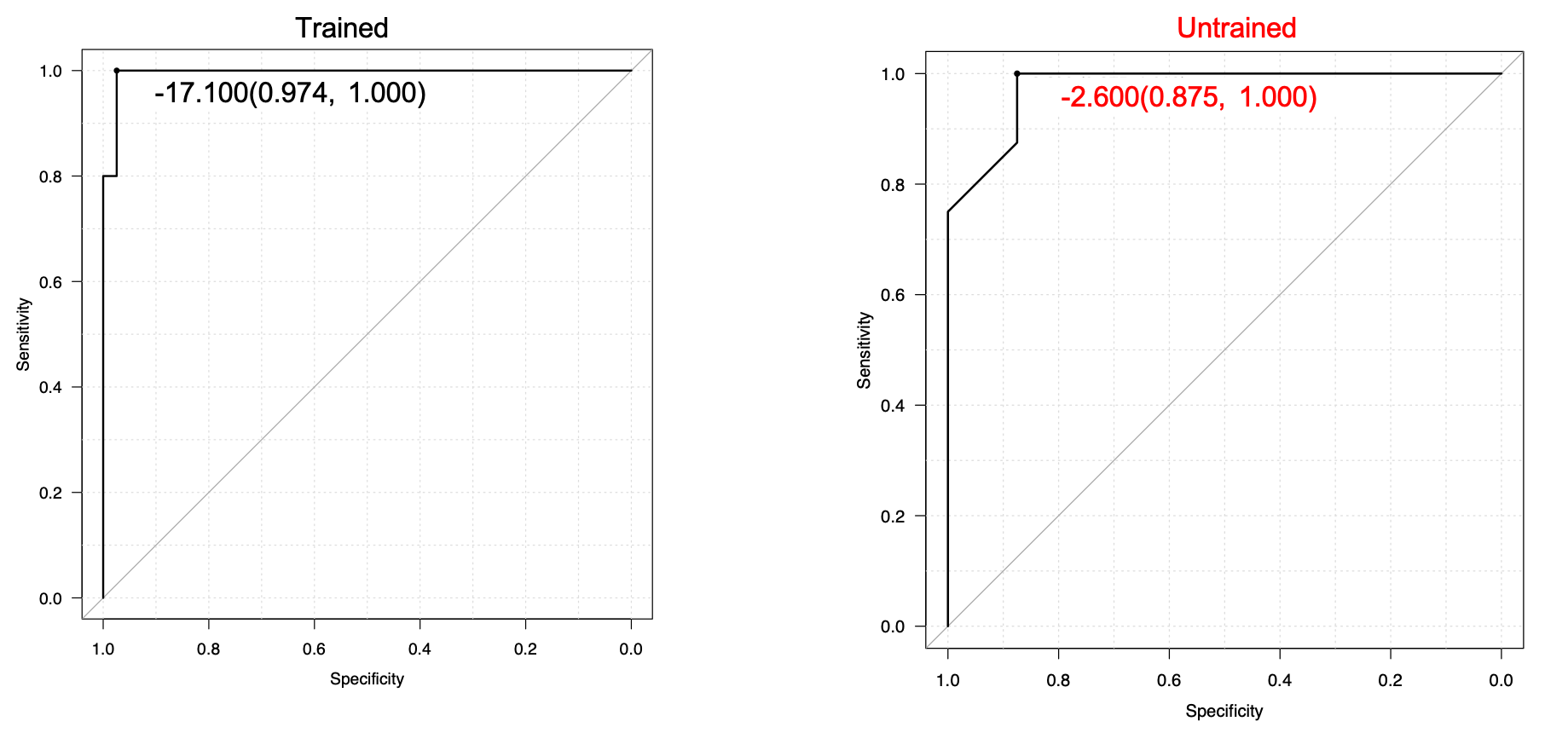


**Online Supplemental Figure 9: Difference between Trained and Un-Trained ΔTOI cutoff values**

The cutoff value for ΔTOI was -17.1 for Trained compared to -2.6 for Untrained.
